# Harnessing Local Immunity for an Effective Universal Swine Influenza Vaccine

**DOI:** 10.3390/v9050098

**Published:** 2017-05-05

**Authors:** Elma Tchilian, Barbara Holzer

**Affiliations:** The Pirbright Institute, Woking, Surrey GU24 0NF, UK; barbara.holzer@pirbright.ac.uk

**Keywords:** Swine influenza, local lung immunity, lung tissue resident memory T cells, universal influenza vaccines, heterosubtypic immunity

## Abstract

Influenza A virus infections are a global health threat to humans and are endemic in pigs, contributing to decreased weight gain and suboptimal reproductive performance. Pigs are also a source of new viruses of mixed swine, avian, and human origin, potentially capable of initiating human pandemics. Current inactivated vaccines induce neutralising antibody against the immunising strain but rapid escape occurs through antigenic drift of the surface glycoproteins. However, it is known that prior infection provides a degree of cross-protective immunity mediated by cellular immune mechanisms directed at the more conserved internal viral proteins. Here we review new data that emphasises the importance of local immunity in cross-protection and the role of the recently defined tissue-resident memory T cells, as well as locally-produced, and sometimes cross-reactive, antibody. Optimal induction of local immunity may require aerosol delivery of live vaccines, but it remains unclear how long protective local immunity persists. Nevertheless, a universal vaccine might be extremely useful for disease prevention in the face of a pandemic. As a natural host for influenza A viruses, pigs are both a target for a universal vaccine and an excellent model for developing human influenza vaccines.

## 1. Introduction

Influenza A virus infection is a global health threat to livestock and humans, causing substantial mortality and morbidity. H1N1 and H3N2 influenza viruses are endemic in pigs and humans in addition to H1N2 in pigs. Since human origin viruses, or viruses containing human origin gene segments, frequently adapt to transmit efficiently in pigs [1,2], the pig is a source of new viruses capable of initiating epidemics or pandemics in humans of mixed swine, human, and avian origin [3]. As both pigs and humans are readily infected with influenza A viruses of similar subtype, the pig is a robust and appropriate model for investigating both swine and human disease. Like humans, pigs are outbred, and physiologically, anatomically, and immunologically similar to humans. The porcine lung also resembles the human in terms of its tracheobronchial tree structure, lung physiology, morphology, and distribution of receptors bound by influenza viruses [4,5].

Swine influenza virus (SI) infection exhibits a spectrum of clinical signs, ranging from inapparent disease to fever, with overt respiratory signs and disease severity is increased significantly by secondary bacterial infection. SI contributes to sub-optimal reproductive performance and is occasionally associated with fever-induced abortion in sows. Immunisation may be a cost effective control measure to combat SI, but the rapid evolution of the virus is a major obstacle SI diversity is reviewed in [1,6,7]. Not all countries with SI use vaccines to control disease. Current UK policy does not involve immunisation against SI, although it is used in some European countries and widely in the US. While it will be difficult to convince government and livestock keepers to invest in control measures for a disease causing insidious losses without providing clear economic and welfare benefits, with pig production intensifying worldwide it is likely that an improved immunisation strategy for SI would result in more countries relying on vaccines to control disease.

Parenterally-administered inactivated vaccines against SI, widely used in the US, are strain-specific and protection correlates with the presence of neutralising antibodies. The most commonly used are whole inactivated virus (WIV) administered with oil-in-water adjuvants, non-replicating alphavirus RNA particle, or autogenous vaccines. For autogenous vaccines the vaccine organism(s) must come from the herd in which the vaccine is to be used, and they accounted for more than half of all SI vaccine doses released for sale in 2008. Recent reviews by Sandbulte et al. [8] and Vincent et al. [6] provide comprehensive information on currently used vaccines in the field in the US. In Europe, RESPIPORC FLU 3 (IDT Biologika, Dessau-Rasslau, Germany) and Gripovac (Merial, Lyon, France) against H1N1, H1N2, and H3N2 circulating SI viruses are used in approximately 5–25% of the pig farms in Belgium, Denmark, France, Spain, Italy, Poland, and Germany.

However, these vaccines do not protect against new viral strains and show poor efficacy in the field because of the evolution of the virus [9]. This lack of efficacy against mismatched strains has two consequences: (1) it requires frequent reformulation and production of influenza vaccines based on the prediction of strains that may circulate. While virological surveillance for human influenza A and B viruses is the cornerstone of the World Health Organisation vaccine selection process, a similar strategy for pig (and other) animal influenza vaccines is still lacking; and (2) in the event of the emergence of a completely novel reassortant virus, there is little or no efficacy leaving both swine and humans at high risk of infection with potential for pandemic spread. A further problem of WIV with oil-in-water adjuvants is enhanced respiratory disease and increased pathology, associated with immune complexes of low avidity or non-neutralising antibodies. Vaccine-associated enhanced respiratory disease (VAERD) has been observed in pigs when heterologous SI infection occurs after immunisation with mismatched WIV [10,11,12,13]. WIVs are also commonly used in pregnant sows to prevent SI infection in piglets [14]. In these circumstances, maternally derived antibodies can be detected in piglets up to 14 weeks after birth and levels correlate with protection against homologous infections [12]. However, after heterologous challenge WIV induced maternally-derived antibodies were associated with enhanced clinical signs [15].

## 2. Universal Influenza Vaccine/Heterotypic Immunity

A cross-protective “universal vaccine” would be an enormous advantage in preventing SI in pigs and reducing the zoonotic threat. The development of a “universal” or broadly protective influenza A vaccine depends on inducing immune responses to conserved components of the virus that either prevent infection or limit replication of virus after infection has occurred. The classical broad immunity detected in convalescent animals and humans is dominated by the latter, mediated by cross-reactive CD4 and CD8 T lymphocytes [16]. The phenomenon was first observed after the isolation of human influenza in the 1930s when ferrets that had recovered from swine influenza were immune to the human virus but did not make detectable cross-neutralising antibodies [17,18]. The term “partial heterotypic immunity” (also called “heterosubtypic”) was introduced by Schulman and Kilbourne in 1965 who observed that mice infected with a H1N1 strain and subsequently challenged with a lethal H2N2 virus, had reduced viral titers in the lung, milder lung pathology, and decreased mortality in the absence of neutralizing antibodies [18]. Since then, studies in multiple animal models including non-human primates have observed reduction in viral load, lung pathology, weight loss and decreased mortality, but not infection, in the absence of detectable cross-neutralizing antibodies (reviewed in [19,20]).

Experimental infection and recovery from SI infection in pigs has also been shown to completely or partially protect against infection with another type of SI [21,22,23,24]. Post-infection immunity to H1N1 and/or H3N2 viruses conferred cross protection against H1N2 in pigs [23], in the absence of detectable haemagglutination inhibition and virus neutralising antibodies, although inhibitory anti-neuraminidase (NA) antibodies were detected prior to infection with H1N2. In contrast a double vaccination with an inactivated H1N1 and H3N2 based vaccine did not confer significant cross-protection [25], highlighting the importance of active virus replication for the induction of heterosubtypic immunity in pigs, directed towards the conserved internal proteins [26]. In line with these findings partial protection was observed in pigs with infection-induced immunity against avian-like H1N1 upon challenge with H3N2, whereas pandemic (pdm)H1N1-immune pigs were not protected because the pdmH1N1 has a different set of internal genes from the challenge strain [22]. In another study pigs previously infected with H1N1, and subsequently challenged with H3N2, did not get fever, showed reduced virus shedding, and showed no transmission to contact pigs [21]. The observed heterosubtypic protection occurred in the absence of cross-reactive haemagglutination inhibition antibodies after primary infection, but correlated with increased serum immunoglobulins (Ig)G antibody levels against the conserved extracellular domain of matrix protein 2 (M2) and nasal nucleoprotein (NP)-specific IgA antibodies. In addition, increased numbers of CD8 T cells were observed in the lungs of pigs previously infected with H1N1 and challenged with H3N2. The extent of heterosubtypic protection varies greatly between studies partly because of the immunisation/challenge strategies used, as well as the degree of conservation of the internal genes between the strains used for immunisation and challenge.

In this review we shall discuss the importance of local lung immunity and the feasibility of making an effective SI universal vaccine.

## 3. Respiratory Tract Immunisation

For optimum induction of heterotypic immunity in experimental animals, virus infection of the lung is required, as opposed to infection of the upper respiratory tract or other peripheral sites [27,28]. Immunisation via the respiratory tract has been shown to be a highly effective means of immunising against influenza. A recent murine study that evaluated the capacity of inactivated influenza vaccine or live attenuated influenza vaccine (LAIV) to induce protective lung response showed that site specific productive infection is required. Interestingly intra-nasal administration of inactivated vaccine or parenteral administration of LAIV failed to elicit protective T cell responses, confirming the requirement for respiratory targeting of LAIV to establish cross-protection [29]. Experimentally, immunisation via the respiratory tract is also highly protective against several other pulmonary diseases in livestock, including bovine tuberculosis [30,31], respiratory syncytial virus in cattle [32] and porcine reproductive and respiratory syndrome virus [33].

Although it is clear that local mucosal immunity is critical for protection against respiratory tract diseases in most cases it is not known what part of the respiratory tract should be targeted to induce optimal protection. Even in non-human primates and humans, where a common respiratory mucosal system has been postulated [34,35], so that immunisation of any part of the respiratory tract might be expected to protect the whole tract, it is not clear whether immunisation of the upper (URT) or lower respiratory tract (LRT) is more effective. A number of studies in mice and ferrets with influenza vaccines show that the LRT is the best target for inhaled influenza vaccination [36,37]. Targeting the LRT and URT by inhalation with the candidate universal influenza vaccine S-FLU (a non-replicating, pseudotyped influenza virus where the viral RNA (vRNA) encoding the haemagglutinin (HA) was inactivated by suppression of the HA signal sequence) resulted in a high degree of cross-protection against H1 and H3 influenza strains in mice [38]. This confirms the studies of Lau et al. [27] showing that protection of mice from H5N1 influenza by LAIV requires delivery of vaccine to the lung as opposed to the URT alone. Neutralising Ab were not induced by S-FLU immunisation of mice, ferrets, or pigs [38,39,40], but strong local lung cellular immune responses were detected, which correlated with protection against challenge. S-FLU coated with H1 or H5 HA has also been administered to pigs to the LRT by aerosol or intra-tracheal methods [40]. The aerosol method was the most efficient in reducing virus titre in nasal swabs and lung tissue after live virus challenge with pandemic H1N1 virus in pigs [40]. Similar comparisons between intra-tracheal and aerosol delivery have been performed with adenoviral vectored TB [30] or Ebola vaccines [41] in non-human primates and, in both cases, aerosol delivery offered superior protection compared to other mucosal routes.

## 4. Live Attenuated Influenza Vaccines

Currently, cold-adapted LAIVs administered by nasal spray, targeting the URT, have been approved for equine (FluAvert, MSD Animal Health, Milton Keynes, UK) and human species (FluMist/Fluenz, MedImmune Gaithersburg, MD, Maryland, US). Studies in young children suggest that LAIV is more protective than inactivated influenza vaccines in those not previously exposed to influenza or influenza vaccines, due to increased vaccine-induced T cell and/or secretory IgA responses. In adults with extensive and partially cross-reactive pre-existing influenza immunity, LAIV boosting of secretory IgA reactive with HA and non-HA antigenic targets expressed by circulating influenza strains, may be an important additional mechanism of vaccine-induced immunity [42]. FluMist was introduced for infants in the UK in 2013, although because of troubling results of recent effectiveness studies, it will not be used during the 2016–2017 vaccination season in the US. The reason for the apparent decreased effectiveness of LAIV as compared with the efficacy shown in the original studies is unclear [43], but it could be related to repeated immunisations and increased baseline immunity, which might interfere with vaccine-virus replication [44].

Experimental LAIV vaccines delivered by the intranasal route have been shown to be protective in pigs [45,46,47,48]. Eight segment SI virus harbouring two different HA (H1 and H3) was generated by replacement of the ectodomain of the NA with the ectodomain of a second HA (H3), thus creating a virus displaying two different HAs (H1 and H3) on the surface [49]. The resulting vaccine was attenuated in pigs and conferred reduction of fever and other clinical signs as well as decreasing gross lesions in the lungs after challenge with both H1 and H3 SI viruses. LAIVs carrying an elastase cleavage site [50,51], NS1 truncations [24], or temperature-sensitive mutations in the polymerase basic protein (PB) 2 and PB1 segments [45,47] all provide degrees of cross-protection after challenge with antigenically distinct viruses but from the same subtype. Sterilizing immunity with no transmission to naïve pigs, was achieved with the temperature sensitive LAIV as opposed to the NS1 truncated LAIV where transmission was not prevented [45]. Considerably high IgA antibody responses in nasal washes and bronchoalveolar lavage (BAL) against whole virus were found in LAIV-immunised pigs compared to inactivated vaccine groups. The elevated IgA levels alone did not determine sterilizing immunity as both tested LAIV vaccines induced equal titers, but only one prevented transmission, suggesting additional requirements for protection, such as induction of cell-mediated immunity [45]. Partial heterosubtypic protection with the LAIV vaccine carrying the elastase cleavage site was observed when the vaccine was administered in a prime/boost regime and challenged either intratracheally [50] or intranasally [46]. As in other studies with LAIV vaccines, cross reactive IgG in serum and IgA in BAL and nasal mucosa were detected. IFNγ secretion by lymph node cells could be induced by exposure to the heterologous challenge strain, suggesting that cell-mediated immunity was also involved in the cross protective effect. Similarly, in NS-1-truncated LAIV immunised animals, heterosubtypic T cell priming against the challenge strain was observed [24], however immune responses waned at 7–8 weeks post immunisation and recall responses of LAIV-immunised pigs were in general lower when compared to wild type-infected pigs or not different from naïve pigs. These results suggest that although LAIV offer partial cross-protection it is less efficient than infection with a live virus.

Clearly the route of delivery is very important for induction of local immunity. Intranasal delivery of a LAIV vaccine was more efficient in inducing mucosal antibodies when compared to intramuscular delivery [48]. It may also be that intra-nasal delivery in pigs is effective because a small proportion of the administered dose reaches some of the lung lobes, albeit most of the material delivered intra-nasally is deposited in the stomach and oesophagus [52]. Whether LAIVs will have much enhanced protective efficacy if deliberately delivered to the LRT requires thorough investigation to determine whether this is more efficient in inducing both homologous and heterologous protection against SI in pigs. However, despite major advances in aerosol vaccine delivery in humans [53] and some examples of local delivery to livestock, no practical devices for aerosol delivery are yet available for farm animals in the field.

## 5. Safety and Danger of Re-Assortment of LAIV

LAIV given as a large droplet aerosol to the URT in humans still requires updating to keep pace with antigenic drift, implying that type specific antibody is the main mechanism of protection. By contrast when administered to the LRT in mice and ferrets it can act as a “universal” influenza vaccine by inducing a multi-component response that is cross-protective between group I (H5N1) and group 2 (H7N9) influenza viruses [39,54]. However, administration of LAIV by small droplet aerosol to the lung in humans (or other species) is strongly discouraged by the manufacturer [55], due to concerns that replication is not completely restricted at 37 °C. In addition pre-pandemic versions of LAIV contain full length versions of vRNAs encoding potentially pandemic HAs, which could reassort into seasonal influenza viruses in cases of dual infection. Similarly reassortment of LAIVs with wild-type swine strains is possible. Furthermore, mixing of different vaccines from different sources is common in the field and if two LAIV vaccines were to be mixed prior to administration to pigs, there is a chance that a wild type SI will be generated with gene segments derived from unmodified gene segments in each LAIV vaccine. This might lead to even more diversity of swine IAV strains or strains with increased zoonotic potential [6].

The universal vaccine candidate, S-FLU was designed to overcome these objections. It is based on the suppression of HA signal sequence, most of the coding sequence of the HA viral RNA is deleted. HA protein is provided from a transfected cell line by pseudotyping. This design allows infection by S-FLU to occur once only and replication of vaccine in the lung or nose is prevented, but all of the conserved viral proteins are expressed in the cytosol of S-FLU infected cells and available for antigen presentation to T lymphocytes [56]. S-FLU cannot replicate in the lung due to lack of a viable HA vRNA, and cannot donate a viable vRNA encoding HA because it does not contain any genetic information from the potentially pandemic virus, being only coated in HA protein. Another single-replication cycle universal vaccine candidate RedeeFlu (FluGen Inc, Madison, WI, USA) is based on the partial deletion of the M2 gene [57] and shown to elicit humoral, mucosal, and cell mediated immunity. The vaccine induced sterilising immunity to homosubtypic challenge, but although it protected mice against lethal heterosubtypic challenge it did not prevent viral replication in nasal turbinates and lung.

## 6. Immunological Mechanisms of Heterotypic Protection

Investigation of the immunological mechanisms mediating the cross protective heterotypic immunity in animal models revealed the role of CD8 and CD4 T-cells and has been reviewed in [20]. The specificity of the protective effect correlates with the conserved viral core antigens recognised by T cells [16,58,59] and protection can be transferred in mice with core protein-specific T cells, particularly class I restricted cytotoxic T lymphocytes [60,61]. A recent prospective study in humans also showed this correlation between cross-reactive CD8 T cell responses and protection from symptomatic infection during the H1N1 influenza pandemic [62,63], confirming earlier work from experimental challenges with influenza virus in humans [59,64].

Similar CD8 systemic responses against the internal NP and M proteins were induced in both pigs and humans after intra-muscular immunisation with a candidate universal Modified Vaccinia Ankara vaccine encoding NP and M1 proteins [65]. This vaccine has been tested in co-administration regimes with either HA protein or following prime boost with a chimpanzee adeno vector also expressing NP and M1 and was shown to induce T cell responses to NP and M1 in pigs. However, no challenge was performed. Whether CD8 cells induced by parenteral immunisation would have the same protective effect as CD8 cells induced by local pulmonary infection or immunisation remains to be established, although there are ongoing efforts to induce mucosal responses through systemic parenteral immunisation in other diseases [66,67]. Alternatively harnessing simultaneously both local and systemic immunity might be an optimal strategy as immunisation against tuberculosis in mice and non-human primates has demonstrated [68,69].

However, despite the wealth of evidence for both CD4 and CD8 T cell mediated cross-protection in mice and humans, studies in pigs are very scarce. An immunoinformatic tool for predicting swine T cell epitopes identified highly conserved class I and class II epitopes among seven SI strains prevalent in US pig herds. The class I peptides were restricted to the external proteins, while responses to class II peptides were focused on epitopes derived from the internal proteins [70]. This is in contrast to humans in whom most cross-reactive CD8 and CD4 T cell epitopes are derived from internal influenza virus proteins. SI class I epitopes were also identified and tetramers developed in pigs following four repeated immunisations with different SI strains in incomplete Freund’s adjuvant [71]. Further studies will be needed to evaluate whether epitope based vaccines will be able to reduce viral burden and morbidity.

The only studies analysing local and systemic immune responses following infection with SI by the pulmonary route, albeit intra-tracheal infection with high dose H1N2, revealed multifunctional T cells with diverse cytokine profiles and in vitro reactivity against heterologous influenza strains, supporting their potential to combat heterologous influenza virus infections in pigs [72,73]. Low frequency SI specific IFNγ producing CD4 and CD8 cells were detected in the lungs four days post-infection, reaching a peak at nine days post infection. At six weeks post-infection CD4 and CD8 memory T cells had accumulated in the lung tissues, but it is not known which epitopes these cells are targeting. Neither is it known whether natural infection induces cells which recognise the informatically defined epitopes.

## 7. Tissue-Resident Memory T Cells

T cell memory was previously considered to be mediated by recirculating memory cells able to pass from the blood to tissues and back to the blood via the lymph. It was not thought that the cells remained in tissues for more than few hours, nor that they divide in non-lymphoid tissues. However recent overwhelming evidence indicates the importance of local tissue-resident memory T cells (T_RM_) in protective immunity. T_RM_ are a newly-defined subset of memory T cells generated following primary infection in tissues such as the respiratory tract, gastrointestinal tract and skin and they persist at these sites after the pathogen has been cleared by the immune response [74,75]. Upon subsequent infection, pathogen-specific T_RM_ cells mount a rapid local response that is independent of T cell recruitment from the blood. Surprisingly, sessile T_RM_ greatly outnumber recirculating T cells within non-lymphoid tissues [76]. They express homing molecules such as the integrin CD103 and the activation marker CD69, the latter indicating that these cells are dividing and likely express effector function. However, there is some phenotypic heterogeneity and overlap of phenotype with cells in blood and lymphoid tissues, so that T_RM_ are best defined by their inaccessibility from the blood stream. This has been elegantly demonstrated by the intravenous administration of labelled antibody, which identifies all intra-vascular, but not tissue-resident cells [76] and by experiments which prevent efflux of cells from lymph nodes so that recirculating cells cannot reach the tissues [77,78].

It is increasingly evident that lung T_RM_ play a major role in protective immunity against respiratory tract infections. In mice both CD4 and CD8 lung T_RM_, induced by immunisation via the respiratory tract, mediate immunity against heterosubtypic influenza strains, enhancing viral clearance and survival after lethal challenge [29,77,79,80]. Influenza-specific T_RM_ are also present in most healthy humans [81,82]. After pneumonectomy for isolated tumours, histologically normal human lung tissue far from the tumours contains both CD4 and CD8 cells with diverse T cell receptors (TCR) that express CD69, produce TNFα and IFNγ and proliferate in response to influenza virus. T_RM_ were also found in a large survey of human tissues [83] and in BAL after respiratory syncytial virus infection [84].

The effector functions of T_RM_ remain incompletely understood, but may involve: (1) direct killing of pathogen-infected cells; (2) release of cytokines that render the surrounding environment non-permissive for pathogen replication; and (3) promoting recruitment of infection-fighting cells from the circulation [75]. The only studies on the duration of cross reactive immunity mediated by T_RM_ have been performed in mice and have shown that heterosubtypic immunity was substantially reduced after seven months [77].

The few studies that have investigated how T_RM_ cells are induced and maintained have used mouse models. Factors necessary for the induction of T_RM_ are not currently well understood but both the route of antigen encounter and the nature of the antigen itself may play a crucial role [29]. However, despite these advances in mice and although T_RM_ cells have been identified in the human lung, we currently understand very little about the role of T_RM_ in any other species, how they are generated in response to infection or vaccination, or how best to induce them. There is no information on T_RM_ in pigs or other livestock, but it is to be expected that they play an important role in protection against SI in these species.

## 8. Mucosal Associated Invariant T Cells 

Although conventional T cells are clearly important, the mucosal immune system contains other cell populations whose functions are less clear. One such population is the recently discovered mucosal associated invariant T cells (MAITs), which exhibit properties of both innate and adaptive cells, as do natural killer T cells and gamma delta T cells (reviewed in [85,86,87]). They are highly enriched in mucosal sites, including lungs, intestines and liver, but are also present in the periphery where they can make up between 1–10% of the total circulating lymphocytes. In humans, MAITs express the Va7.2-Ja33 chain of the T cell receptor, make up to ~10% of blood CD8 cells and detect riboflavin metabolites bound to the non-polymorphic major histocompatibility complex (MHC) class-I related protein, MR1.

MAITs are associated with protection from bacterial infections, but recently were shown to be activated by influenza virus through mechanisms independent of MR1-TCR engagement and mediated through IL-18 combined with IL-12, IL-15, and/or interferon-α/β, which are expressed by a range of antigen presenting cells and monocytes [88]. The importance of MAITs in influenza is suggested by the correlation of patient survival with a ~2.5 fold increase in total MAITs cell numbers. Here too, MAIT activation was highly dependent on secondary cytokine stimulation derived from monocytes and activation is primarily, but not entirely, IL18-dependent [89]. MAITs have been identified in sheep and cattle, but there is no published data on pigs, nor has the role of MAITs in SI been defined.

## 9. Antibody Mediated Cross-Protective Immunity

Protective humoral responses to influenza are usually associated with antibodies against its surface glycoproteins HA and NA and in the last decade many laboratories identified broadly cross-protective antibodies directed against the highly-conserved HA stalk domain that showed different levels of cross-reactivity towards group 1 [90,91,92], group 2 [93,94,95] or both group 1 and 2 viruses [96,97,98,99]. Multiple studies have shown that in vivo efficacy of broadly cross neutralising anti-stalk antibodies is dependent on Fc-dependent mechanisms, like antibody-dependent cytotoxicity, antibody-dependent cellular phagocytosis, and complement-dependent cytotoxicity. A problem related to the development of anti-stem antibodies is their variable neutralising potency against viruses belonging to different subtypes and the existence of natural escape mutants, although some exhibit unprecedented breadth and potency [100]. Vaccine strategies targeting antibodies to the conserved stalk region of HA involve “headless” HA although responses were suboptimal, probably because removal of the head domain destroyed important conformational epitopes, or destabilized the stem domain in such a way that it was no longer able to elicit protective antibody responses. An alternative approach uses a vaccine consisting of a chimeric HA structure made of the conserved HA stem domain of an H1 or H3 strain of influenza, capped with the HA head domain from an exotic zoonotic influenza strain that has not been previously encountered by the human population, most likely directing the recall responses to stimulate pre-existing stem-reactive memory B cells (reviewed in [101,102]).

A recent mouse study suggests that memory B cells specific for broadly neutralizing epitopes may reside in the lungs instead of in circulation or in secondary lymphoid organs. These lung-resident memory B cells were highly mutated, and were generated as a result of local persistent germinal centres that are responding to prolonged exposure to viral antigens found in the lung. These tissue-resident memory B cells provided robust protection against a drifted virus in a secondary challenge model, confirming their importance in generating a cross-reactive, broadly neutralizing antibody response against influenza [103]. Although these findings have not yet been confirmed in humans or livestock, it does suggest a role for tissue-resident memory B cells in providing broadly-neutralizing responses.

IgA has been implicated in host protection against influenza in several studies [104,105]. Polymeric forms of IgA in nasal washes from adults vaccinated intranasally with a WIV are very potent in neutralizing influenza virus [106]. Interestingly IgA can also intercept antigen within epithelial cells [107] and for example, in that way NP-specific IgA in the nasal mucosa in pigs after SI infection may contribute to virus clearance [21]. HA-specific IgA antibody responses in the nasal washes have been detected as early as four days after intranasal or intratracheal inoculation with SI in 50% of pigs [108]. Cells isolated from the nasal turbinates and lymphoid tissue of the soft palate were tested for their ability to secrete influenza virus-specific IgA or IgG [109]. Notably those cells produced IgA, whereas significantly less cells produced IgG. Most likely IgA-producing cells can also be isolated in pigs from the lungs as high titres of IgA were found in the BAL fluid. In mice protection from influenza correlated with influenza-specific IgA and IgG antibody secreting cells in the lung at the time of challenge [110]. Therefore, it is quite likely that a major contributor to the protective efficacy of LAIV vaccine is IgA produced in the local mucosa.

Recently the breadth and magnitude of Ab response directed against HA and NA has been determined in mice, guinea pigs and ferrets following sequential infections with H1N1 or H3N2 influenza viruses [111]. Guinea pigs developed high titres of broadly cross-reactive antibodies, while mice and ferrets exhibited narrower responses. When these were compared to the antibody responses after infection of humans with H1N1 or H3N2 a markedly broad response was found, with the cross-reactivity profiles dependent on the viral strain first encountered during childhood (original antigenic sin). Re-analysis of the Cleveland Family Study which monitored families before and during the pandemic of 1957 where a subtype shift from H1N1 to H2N2 occurred, did show significantly lower incident rates during the pandemic in adults that contracted symptomatic influenza before, compared to children [112]. Recently it was shown that childhood hemagglutinin imprinting conferred lifelong protection against severe infection and death from novel HA as long as the virus belonged to the same phylogenetic group as the one encountered first in an individual’s life [113]. Better understanding of cross-reactive immunity in humans is important for the development of universal vaccine strategies that are designed to boost pre-existing antibodies to protective levels. However, there is surprising lack of information about the landscape of the antibody response in pigs, which are exposed to multiple SI and very often are infected with one or more different SI subtypes without overt disease, a situation that may reflect better the immune responses in humans with pre-existing immunity and a complicated history of exposure to influenza viruses.

However, the role of antibodies in protection needs further evaluation and caution, because of some evidence for enhancement of disease by antibody. VAERD was associated with the presence of high titre cross-reacting non-neutralizing antibodies targeting the conserved stem HA2 domain at a site adjacent to the fusion peptide. In the absence of neutralizing antibodies against the HA1 globular head of pdmH1N1, HA2 antibodies increased virus infection of MDCK cells in vitro and enhanced membrane fusion [114]. However, VAERD is mainly associated with WIV administered with oil-in-water adjuvant parenterally, and has not been reported after LAIV administration by the mucosal route [115]. Although an immune response against mismatched HA protein alone was enough to cause VAERD [116], it has been shown recently that NA inhibiting antibodies to the homologous NA were sufficient to abrogate it [117]. There clearly is a need to determine the relative contribution of all types of anti-influenza antibodies, rather than focusing only on those to the HA stem.

## 10. Conclusions

The global influenza community is in urgent need of a universal influenza vaccine capable of inducing durable cross-protection against a broad spectrum of influenza virus strains. Harnessing local T cell immunity is essential for the development of a practical and effective universal influenza vaccine. Vaccines that induce T cell mediated immunity reduce disease severity and may reduce or prevent transmission, but do not prevent infection. The evaluation, licensure and deployment of a vaccine not designed to prevent infection, but to limit the severity of disease, will be challenging, as it must show comparable or better efficacy than current vaccines. However, universal vaccines might substantially reduce the severity of infection and limit the spread of disease during outbreaks and could be used ‘off the shelf’ early in an outbreak or pandemic, before strain-matched vaccines are available. In contrast a universal vaccine based on antibody responses would prevent infection and broadly-neutralizing antibodies specific for the conserved stem domain of the HA have shown promise both from a therapeutic perspective, as well as for guiding vaccine design efforts. Since most studies of human broadly-neutralizing monoclonal antibodies have focused primarily on B cells isolated from blood samples, a focus on lung-resident memory B cells may provide greater insight into how to generate broadly neutralizing antibodies. Future delivery strategies must consider how to best boost lung-resident memory B cells to elicit potent broadly protective responses. Combining both approaches would be a viable strategy and only field trials will show whether a universal vaccine could induce sufficient herd immunity to be useful. It is important to be aware of the limitations of the animal models used to study universal vaccines. It is challenging to scale up from 25 g mice to 70 kg humans. Pigs of 10 to 60 kg approach the weight of humans and, therefore, have great potential as a translation tool for therapeutics, such as antibodies and vaccines. Swine are also natural hosts of influenza viruses and play a critical role in the emergence and epidemiology of novel and potentially zoonotic influenza viruses. They are, therefore, a powerful model to study immunity to influenza.
